# Systemic Immune-Inflammation Index as a Predictive Biomarker for Pre-Eclampsia

**DOI:** 10.3390/jcm15103619

**Published:** 2026-05-08

**Authors:** Dimitris Baroutis, Eleni Katsianou, Aikaterini-Gavriela Giannakaki, Nikolaos Sindos, Ioannis Fragiskos, Konstantinos Koukoumpanis, Vasilios Lygizos, Marianna Theodora, Vasilios Pergialiotis, Michael Sindos, George Daskalakis

**Affiliations:** 1 1st Department of Obstetrics and Gynecology, Alexandra Hospital, National and Kapodistrian University of Athens, 11528 Athens, Greecegdaskalakis@yahoo.com (G.D.); 2 2nd Department of Pediatrics, “P. & A. Kyriakou” Children’s Hospital, National and Kapodistrian University of Athens, 11527 Athens, Greece

**Keywords:** systemic immune-inflammation index, pre-eclampsia, biomarker, inflammatory markers, neutrophil-to-lymphocyte ratio, platelet-to-lymphocyte ratio, pregnancy complications, predictive biomarker

## Abstract

Pre-eclampsia, a major obstetric syndrome with an estimated global incidence of 2–8% of all pregnancies, ranks among the foremost causes of adverse maternal and perinatal outcomes worldwide. The Systemic Immune-Inflammation Index (SII)—derived as the product of platelet and neutrophil counts divided by the lymphocyte count—is a composite hematological parameter first established in oncological research that simultaneously captures neutrophil activation, lymphocyte dysfunction, and platelet alterations—three immunohematological disturbances implicated in pre-eclampsia pathophysiology. This narrative review synthesizes the current evidence regarding SII in pre-eclampsia, examining the biological rationale, clinical study findings, comparative performance against established inflammatory biomarkers, practical advantages, and limitations. A comprehensive literature search encompassing PubMed/MEDLINE, Scopus, Web of Science, and Google Scholar was conducted, covering all available records up to January 2026. The available data reveal substantial heterogeneity in both the direction of association (elevated versus paradoxically decreased SII in pre-eclampsia) and diagnostic performance across populations. While some studies report significantly elevated first-trimester SII values in women who subsequently develop pre-eclampsia, others demonstrate lower values or no significant predictive capacity. The only available meta-analysis reported non-significant pooled results for the pre-eclampsia-specific subgroup. Although SII’s derivation from routine complete blood count testing presents the advantages of cost-effectiveness and universal accessibility, methodological limitations—including retrospective study designs, the absence of standardized thresholds, the inconsistent discriminatory performance, and the conflicting directionality of association—preclude clinical implementation at present. Integration within multiparametric prediction models may optimize SII’s clinical utility. Future research should prioritize prospective validation studies across diverse populations, mechanistic investigations, and randomized controlled trials to establish evidence-based clinical translation.

## 1. Introduction

Pre-eclampsia constitutes one of the most clinically significant obstetrical complications, manifesting as new-onset hypertension combined with proteinuria or end-organ dysfunction after 20 weeks of gestation [1,2]. Affecting approximately 2–8% of pregnancies globally, this multisystemic disorder exhibits substantial geographic and demographic variation, with incidence rates demonstrating marked disparities between high-income and low- and middle-income countries [1,3]. The clinical spectrum ranges from mild disease manageable through expectant monitoring to severe manifestations including eclampsia, HELLP syndrome (hemolysis, elevated liver enzymes, and low platelets), and life-threatening complications such as cerebrovascular accidents, pulmonary edema, and acute kidney injury [2,4]. Pre-eclampsia substantially contributes to maternal mortality worldwide, accounting for approximately 14% of maternal deaths globally and representing one of the leading preventable causes of pregnancy-related mortality [5,6]. The disease burden is disproportionately borne by women in low- and middle-income countries, highlighting significant health disparities in maternal care [3,7].

### 1.1. Pathophysiology of Pre-Eclampsia

The pathogenesis of pre-eclampsia is underpinned by multifactorial mechanisms in which deficient extravillous trophoblast invasion and the failure of physiological spiral artery remodeling result in placental hypoxia, oxidative injury, and the consequent liberation of anti-angiogenic and pro-inflammatory mediators into the maternal circulation [8,9]. According to the widely endorsed two-stage pathogenic framework, aberrant placentation (Stage 1) precedes the systemic endothelial injury that characterizes clinical disease manifestation (Stage 2), although considerable heterogeneity exists in specific etiological pathways leading to clinical manifestation [10,11]. The molecular determinants governing trophoblast invasion and spiral artery remodeling—encompassing cytokine networks, growth factor signaling (including VEGF and Notch pathways), and post-translational regulatory mechanisms such as ubiquitination—have recently been further elucidated [12,13], underscoring the molecular complexity underpinning placental establishment in normal pregnancy and its disruption in pre-eclampsia. During normal gestational progression, extravillous trophoblast cells infiltrate and remodel maternal spiral arteries, transforming narrow, high-resistance arterial structures into dilated, high-flow conduits capable of sustaining the escalating hemodynamic requirements of the fetoplacental unit [8,14]. In pre-eclampsia, this vascular remodeling process is compromised, maintaining a narrow, high-resistance arterial architecture [9,10].

Such deficient placental establishment triggers placental oxygen deprivation, tissue ischemia, oxidative cellular damage, and the liberation of multiple factors into the maternal systemic circulation, encompassing anti-angiogenic mediators, pro-inflammatory cytokines, syncytiotrophoblast cellular fragments, and circulating fetal genetic material [10,15]. The disrupted equilibrium between pro-angiogenic mediators (including placental growth factor, PlGF) and anti-angiogenic substances (including soluble fms-like tyrosine kinase-1, sFlt-1, and soluble endoglin, sEng) assumes critical pathogenic importance [16,17]. Increased sFlt-1 concentrations bind circulating PlGF and vascular endothelial growth factor (VEGF), blocking their endothelial cell receptor interactions and triggering endothelial dysfunction [16,18]. This endothelial impairment constitutes the primary pathophysiological foundation of maternal syndrome manifestation, affecting multiple organ systems, including renal, hepatic, and central nervous system function [1,2,8,19].

### 1.2. Classification and Clinical Manifestations

Pre-eclampsia has been traditionally classified based on the timing of onset, with important prognostic and pathophysiological implications [2,8,20]. Early-onset pre-eclampsia (developing before 34 weeks’ gestation) is more often associated with inadequate placentation, severe maternal and fetal complications, and a hemodynamic profile of low cardiac output and high peripheral vascular resistance [20,21]. This subtype is more likely to be associated with fetal growth restriction, reflecting significant placental involvement in its pathogenesis. In contrast, late-onset pre-eclampsia (at or after 34 weeks’ gestation), responsible for the majority of cases (approximately 70%), tends to present with preserved or augmented fetal growth, a hemodynamic profile characterized by variable peripheral resistance, and potentially elevated cardiac output [20,22].

More recently, pre-eclampsia has been subclassified based on the presence or absence of severe features, including severe hypertension (systolic blood pressure ≥ 160 mmHg or diastolic blood pressure ≥ 110 mmHg), thrombocytopenia, impaired liver function, renal insufficiency, pulmonary edema, and new-onset cerebral or visual disturbances [1,2]. The clinical manifestations are multisystemic and reflect widespread endothelial dysfunction. Cardiovascular manifestations primarily involve increased peripheral vascular resistance, producing hypertension, despite the reduced intravascular volume [20]. Renal manifestations most commonly present as proteinuria secondary to glomerular endotheliosis and podocyte injury [4,23]. Hematologic manifestations include thrombocytopenia, hemolysis, and disseminated intravascular coagulation [2,21]. Fetal complications encompass growth restriction secondary to placental insufficiency, preterm delivery, and neonatal morbidity [4,7].

### 1.3. Risk Factors and Prediction

Numerous pre-eclampsia risk factors have been characterized, encompassing specific demographic characteristics (including advanced maternal age, and nulliparity), medical history (including chronic hypertension, pre-existing diabetes, and previous pre-eclampsia), current pregnancy characteristics (including multifetal gestation, and assisted reproductive technology conception), and physiological abnormalities (including obesity, and abnormal uterine artery Doppler velocimetry) [1,2,24]. Contemporary clinical management relies upon risk stratification through the medical history, biophysical assessment, and biochemical evaluation of placental factors, particularly the sFlt-1/PlGF ratio [16,25,26].

Advanced multivariate screening models have demonstrated enhanced detection capabilities. The Fetal Medicine Foundation (FMF) competing risks algorithm—which integrates the maternal history, mean arterial pressure, uterine artery Doppler indices, and angiogenic biomarkers—achieves detection rates of approximately 90% for early-onset and 75% for preterm disease, at a fixed 10% false-positive rate when applied at 11–13 weeks of gestation [24,26,27,28]. However, implementation remains constrained by cost considerations, technical complexity, and limited availability, particularly in resource-constrained healthcare settings where the pre-eclampsia burden is most substantial [7,29].

### 1.4. Prevention Strategies

Among pharmacological interventions, low-dose aspirin represents the most extensively validated and broadly recommended prophylactic strategy for high-risk obstetric populations [30,31]. The landmark ASPRE randomized controlled trial demonstrated that aspirin therapy administered at 150 mg daily from 11–13 weeks through 36 weeks of gestation conferred a greater than 60% reduction in the risk of preterm pre-eclampsia (OR 0.38; 95% CI 0.20–0.74) [30]. Subsequent meta-analyses have corroborated these findings, establishing that the efficacy of aspirin prophylaxis is critically dependent upon treatment initiation no later than 16 weeks of gestation at minimum daily doses of 100 mg [31,32]. Calcium supplementation constitutes an additional evidence-based preventive strategy, yielding a particular benefit among women with insufficient dietary calcium intake [33]. Beyond pharmacological approaches, lifestyle and dietary modifications—encompassing structured exercise regimens, gestational weight management, and nutritional interventions—have been investigated as adjunctive preventive measures, although the quality and consistency of the supporting evidence remain variable across studies [34,35].

### 1.5. Inflammatory Biomarkers in Pre-Eclampsia

Central to pre-eclampsia pathogenesis is the profound dysregulation of maternal immune responses and the activation of systemic inflammatory pathways [36,37]. A physiologically successful pregnancy depends on the active induction of maternal immunological tolerance towards the fetal semi-allograft, mediated through coordinated crosstalk among regulatory T lymphocytes, decidual natural killer cells, and Th2-biased cytokine networks [38,39]. In pre-eclampsia, this immunological equilibrium becomes disrupted, manifesting as excessive pro-inflammatory Th1 and Th17 activation, regulatory T-cell deficiency, aberrant natural killer cell function, and systemic inflammatory cascade activation [40,41].

The recognition that pre-eclampsia fundamentally involves systemic inflammation has stimulated the investigation of diverse inflammatory biomarkers for disease prediction and severity assessment [42,43]. Traditional individual parameters including C-reactive protein, absolute neutrophil count, and platelet indices have demonstrated associations with pre-eclampsia but exhibit a limited discriminatory capacity when employed as standalone markers [44]. Composite inflammatory indices combining multiple hematological parameters have consequently emerged as potentially superior biomarkers. Among composite hematological indices, the neutrophil-to-lymphocyte ratio (NLR) and platelet-to-lymphocyte ratio (PLR) have accumulated the largest evidence base in the pre-eclampsia literature [45,46,47,48], with the meta-analytic pooling consistently documenting significantly higher values in affected women relative to normotensive controls, albeit with considerable between-study variability in discriminatory capacity [46,47,48].

### 1.6. Systemic Immune-Inflammation Index: Overview and Potential Application

The Systemic Immune-Inflammation Index (SII), calculated as platelet count × neutrophil count/lymphocyte count, represents a further evolution in composite inflammatory biomarkers, originally developed and validated in oncology literature for the cancer prognosis assessment [49]. Hu and colleagues first described SII in 2014, demonstrating its superior prognostic value compared to individual blood cell parameters or simpler two-component ratios in predicting survival among patients undergoing curative resection for hepatocellular carcinoma [49]. Subsequent investigations have validated SII’s prognostic significance across diverse malignancies including gastric, colorectal, lung, and pancreatic cancers, as well as in cardiovascular disease, cerebrovascular disorders, and autoimmune conditions [50,51,52,53,54].

The theoretical advantage of SII over simpler two-component ratios stems from its simultaneous incorporation of three fundamental cellular components of systemic inflammation and immune response—neutrophils, reflecting inflammatory cell activation, lymphocytes, representing adaptive immune function, and platelets, indicating thrombogenic potential and inflammatory amplification [50,55]. The application of SII to pre-eclampsia prediction represents a logical extension given that this index captures multiple pathophysiological processes known to be dysregulated in the disease: neutrophil activation and excessive oxidative stress generation, lymphocyte dysfunction with regulatory T-cell deficiency and pro-inflammatory T helper cell expansion, and platelet activation with enhanced thrombogenic activity [56,57,58]. Moreover, SII’s derivation exclusively from complete blood count parameters—a universally obtained, inexpensive, and rapidly available test—presents significant practical advantages for clinical implementation, particularly in resource-limited settings [59,60].

**Figure 1:**
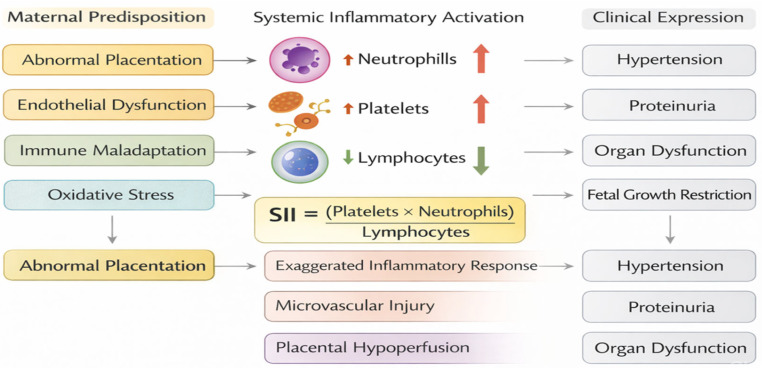
Conceptual framework linking maternal predisposing factors, systemic inflammatory activation, and the Systemic Immune-Inflammation Index (SII) to the biological effects and clinical manifestations of pre-eclampsia. SII = Platelets × Neutrophils/Lymphocytes.

Figure 1 illustrates the pathogenic continuum from maternal predisposition to clinical manifestation, positioning SII as a composite biomarker that integrates the key hematological alterations involved.

This comprehensive narrative review aims to critically evaluate the current evidence regarding SII as a predictive and diagnostic biomarker in pre-eclampsia, examining the biological rationale, clinical study findings, comparative performance against established inflammatory biomarkers, practical advantages, inherent limitations, and future research priorities essential for evidence-based translation.

## 2. Materials and Methods

**Figure 2:**
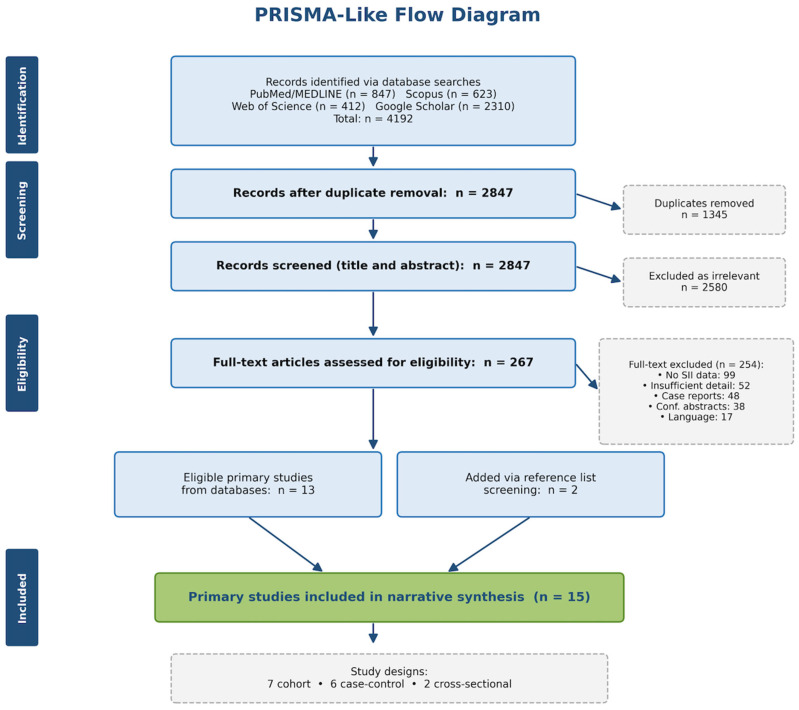
Flow diagram of the literature search and study selection process. Of 4192 records identified across four databases, 15 primary SII–pre-eclampsia studies were included in the narrative synthesis (13 from database screening; 2 from reference list screening), comprising 7 cohort, 6 case-control, and 2 cross-sectional studies. Per-database counts are approximate estimates.

The present work constitutes a narrative review designed to appraise and integrate the available evidence on SII as a biomarker in the pre-eclampsia context. Although a disciplined search and selection framework was applied throughout, adherence to PRISMA reporting standards is not formally required for narrative reviews; however, in the interest of methodological transparency, a PRISMA-like flow diagram documenting the literature identification, screening, and inclusion process has been incorporated as Figure 2.

### 2.1. Search Strategy

Electronic searches were conducted across four databases—PubMed/MEDLINE, Scopus, Web of Science, and Google Scholar—from database inception to January 2026. The primary search string combined terms for hypertensive pregnancy disorders (Preeclampsia OR Pre-eclampsia OR toxemia OR eclampsia OR “gestational hypertension” OR “hypertensive disorders of pregnancy”) with SII-related terminology (“systemic immune-inflammation index” OR “SII” OR “immune inflammation index”). Supplementary searches were performed using terms including “neutrophil-to-lymphocyte ratio,” “platelet-to-lymphocyte ratio,” “inflammatory biomarkers,” and “pregnancy complications.” Reference list screening of retrieved articles served as an additional source identification method.

### 2.2. Study Selection

Inclusion criteria comprised the following: (1) original research articles investigating the association between SII and pre-eclampsia, encompassing case–control studies, cohort studies (prospective and retrospective), cross-sectional investigations, and diagnostic accuracy studies; (2) studies focusing on pregnant women; (3) studies reporting SII values in pre-eclampsia versus control groups; and (4) systematic reviews and meta-analyses examining SII in pre-eclampsia. Studies examining SII in relation to other pregnancy complications (gestational diabetes mellitus, pregnancy loss, and HELLP syndrome) were included when they provided comparative data relevant to pre-eclampsia. Foundational literature on pre-eclampsia pathophysiology, immune dysfunction, and other inflammatory biomarkers (NLR, and PLR) was incorporated to provide comprehensive contextual background. Exclusion criteria comprised case reports, conference abstracts without full-text availability, editorials, and studies lacking sufficient methodological detail for critical appraisal. Only English-language publications were considered. The search was last updated on 31 January 2026. The overall literature search and study selection process is summarized in Figure 2.

### 2.3. Data Extraction and Synthesis

Data extraction was performed independently by two investigators (D.B. and E.K.) using a pre-specified standardized form. Variables retrieved included author, publication year, country of origin, study design, participant characteristics and sample size, gestational age at SII measurement, pre-eclampsia diagnostic criteria, SII values in cases and controls, optimal cut-off values, receiver operating characteristic (ROC) metrics (area under the curve, sensitivity, and specificity), head-to-head comparisons with other biomarkers, and documented confounders. Data were synthesized narratively, organized by thematic categories including first-trimester predictive studies, diagnostic studies at time of presentation, severity assessment, comparative biomarker performance, and population-specific variations.

### 2.4. Quality Assessment

Although the present work constitutes a narrative review and is not subject to PRISMA reporting requirements, a structured methodological appraisal was applied to all included primary studies. Observational studies (cohort and case–control designs) were appraised using the Newcastle–Ottawa Scale (NOS), which evaluates three domains: selection of study groups (maximum four stars), comparability of groups (maximum two stars), and ascertainment of outcome or exposure (maximum three stars). Studies achieving an NOS score of seven or more stars were considered to be at low risk of bias; those scoring five to six stars were classified as moderate-risk; and those scoring fewer than five stars were classified as high-risk. The sole meta-analysis included was appraised using the AMSTAR-2 tool. The overall certainty of evidence for key conclusions was assessed using the Grading of Recommendations, Assessment, Development, and Evaluations (GRADE) framework, which classifies evidence as High, Moderate, Low, or Very Low based on study design, risk of bias, inconsistency, indirectness, imprecision, and publication bias. Given that all primary studies are retrospective observational investigations with small-to-moderate sample sizes, high heterogeneity, and variable control of confounding, the overall GRADE certainty for SII as a pre-eclampsia biomarker was rated Very Low to Low across all outcomes; these ratings are indicated throughout the Discussion where key conclusions are drawn. Additional dimensions appraised encompassed SII derivation methodology, gestational age at measurement, pre-eclampsia diagnostic criteria applied, and adequacy of covariate adjustment. The findings of this appraisal directly shaped the interpretation of results and the characterization of evidence limitations presented in the Discussion.

## 3. Biological and Pathophysiological Basis of SII in Pre-Eclampsia

### 3.1. Neutrophil Activation and Dysfunction

Neutrophils, constituting the most abundant circulating leukocytes, assume critical and multifaceted roles in pre-eclampsia pathophysiology through mechanisms involving excessive activation, dysregulated trafficking, enhanced reactive oxygen species (ROS) production, and the formation of neutrophil extracellular traps (NETs) [61,62]. Normal pregnancy involves moderate neutrophil activation reflecting physiological adaptations supporting the maternal defense against pathogens while accommodating fetal allograft tolerance [63]. In pre-eclampsia, however, neutrophil activation becomes markedly exaggerated and pathological, contributing to systemic endothelial dysfunction, vascular inflammation, and organ damage [61,64]. Women with pre-eclampsia demonstrate a significantly elevated expression of neutrophil activation markers including CD11b and CD64, with the activation intensity correlating with disease severity [64,65].

Activated neutrophils in pre-eclampsia generate excessive quantities of ROS through NADPH oxidase activation and myeloperoxidase activity, overwhelming the maternal antioxidant defense mechanisms and resulting in widespread oxidative stress that damages endothelial cells, promotes lipid peroxidation, and compromises nitric oxide bioavailability [66,67]. Furthermore, NET formation (NETosis) has emerged as an important mechanism in pre-eclampsia pathophysiology [62,68]. NETs consist of extracellular chromatin scaffolds decorated with histones and antimicrobial proteins, and, while physiological NET formation contributes to pathogen defense, excessive or dysregulated NETosis causes endothelial damage, promotes thrombosis, and amplifies inflammatory responses [62,69]. Women with pre-eclampsia demonstrate elevated circulating NET biomarkers, with NET levels correlating with disease severity [63,66].

### 3.2. Lymphocyte Dysfunction and Regulatory T-Cell Deficiency

The lymphocyte compartment undergoes substantial alterations in pre-eclampsia, with the most consistently documented finding being a reduction in the peripheral lymphocyte count (lymphocytopenia) and the qualitative dysfunction of lymphocyte subsets [38,39,40,41]. Regulatory T cells (Tregs), which express the transcription factor FoxP3 and produce immunosuppressive cytokines including IL-10 and TGF-β, play essential roles in maintaining the maternal–fetal immune tolerance by suppressing excessive maternal immune responses against fetal alloantigens [38,39]. The pooled evidence from meta-analyses has repeatedly demonstrated diminished circulating Treg proportions in women with pre-eclampsia relative to healthy normotensive comparators [40,70].

Concurrently, pro-inflammatory T helper cell subsets demonstrate expansion in pre-eclampsia. Th1 cells producing interferon-γ and tumor necrosis factor-α exhibit an increased prevalence, while Th17 cells producing interleukin-17 demonstrate activation and expansion [40,41,71]. The resulting Th1/Th2 and Th17/Treg imbalances create a pro-inflammatory immunological milieu that promotes endothelial dysfunction, complement activation, and vascular inflammation [36,71]. Natural killer (NK) cell dysfunction also contributes, with decidual NK cells exhibiting altered cytokine profiles and cytotoxic potential in pre-eclampsia [72,73]. The lymphocyte component of SII (as the denominator) inversely reflects these immune regulatory deficits, such that lymphocytopenia amplifies the SII value, potentially capturing the degree of immune dysregulation [49,55].

### 3.3. Platelet Activation and Thrombogenic Mechanisms

Platelet abnormalities in pre-eclampsia encompass both quantitative and qualitative alterations reflecting the disease’s prothrombotic state and systemic endothelial dysfunction [56,57]. Thrombocytopenia, occurring in approximately 15–20% of women with pre-eclampsia, results from increased peripheral consumption through platelet aggregation and microthrombus formation, enhanced sequestration in the hepatic and placental vasculature, immune-mediated destruction, and impaired megakaryopoiesis [56,74]. Beyond quantitative changes, platelets in pre-eclampsia demonstrate qualitative abnormalities reflecting chronic activation, with multiple activation markers including P-selectin and activated glycoprotein IIb/IIIa exhibiting upregulation [57,75].

Activated platelets release bioactive molecules stored in granules including adenosine diphosphate, serotonin, thromboxane A2, and platelet-derived growth factor, which amplify inflammatory responses, promote vasoconstriction, and contribute to endothelial dysfunction [56,76]. The mean platelet volume (MPV), which reflects the platelet size and serves as a surrogate for the platelet activation status and marrow turnover, is consistently elevated in pre-eclamptic women [57,77]. Particularly important are platelet–leukocyte interactions, wherein activated platelets adhere to neutrophils through P-selectin binding, forming heterotypic aggregates that amplify inflammatory responses through multiple mechanisms [76,78]. Women with pre-eclampsia demonstrate significantly elevated circulating platelet–neutrophil aggregates, with the levels correlating with disease severity [78,79].

### 3.4. Integrated Rationale for SII in Pre-Eclampsia

The Systemic Immune-Inflammation Index, by simultaneously incorporating the neutrophil, platelet, and lymphocyte parameters in a single mathematical formulation (platelet count × neutrophil count/lymphocyte count), was originally proposed to capture multiple interconnected pathophysiological processes dysregulated in pre-eclampsia more comprehensively than individual parameters or simpler two-component ratios [49,55]. This mechanistic rationale, while conceptually coherent, is derived from pathophysiological inference rather than direct empirical validation in the pre-eclampsia context and must therefore be interpreted with appropriate caution. The numerator (platelet × neutrophil) reflects the magnitude of thrombotic and inflammatory activation, with elevated values indicating the increased platelet activation, the neutrophil-mediated inflammatory response, and their synergistic interaction through platelet–neutrophil aggregate formation. The denominator (lymphocyte count) inversely correlates with the immune regulatory capacity, such that lymphocytopenia—reflecting Treg deficiency and stress-induced lymphocyte apoptosis—amplifies the SII value. Consequently, SII elevation in pre-eclampsia would, if the theoretical framework were borne out empirically, reflect a composite measure encompassing the pro-thrombotic state (elevated platelets), excessive inflammatory activation (elevated neutrophils), and impaired immune regulation (reduced lymphocytes) [49,50,55]. As detailed in Section 4, however, this theoretical construct is not consistently supported by the clinical data.

However, it is important to note that this theoretical framework predicts SII elevation in pre-eclampsia, whereas the empirical evidence, as discussed in Section 4, reveals a more complex and heterogeneous picture that does not consistently conform to this prediction.

**Figure 3:**
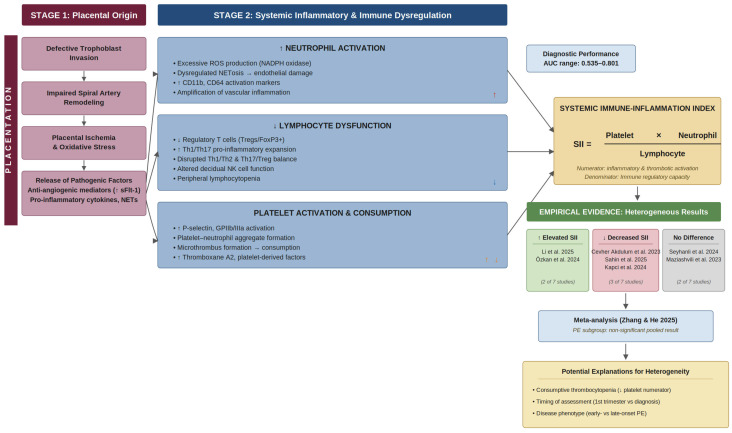
Pathophysiological mechanisms linking the Systemic Immune-Inflammation Index (SII) to pre-eclampsia. The diagram illustrates how defective trophoblast invasion and spiral artery remodeling lead to placental ischemia, triggering inflammatory cascades that affect neutrophil, platelet, and lymphocyte counts, thereby influencing SII values. Reported diagnostic performance across primary studies spans an AUC range of 0.535–0.801 [59,60,80,81,82]. Note that empirical data show heterogeneous SII responses, with both elevated [59,80] and paradoxically decreased [60,81,82] SII reported in pre-eclampsia, while other studies have found no significant difference between cases and controls [83,84]. The only available meta-analysis to date reported a non-significant pooled result for the pre-eclampsia-specific subgroup [85].

Figure 3 summarizes the pathophysiological pathways through which SII components are altered in pre-eclampsia and the heterogeneous empirical findings reported across studies.

## 4. Clinical Studies of SII in Pre-Eclampsia

The literature examining SII in relation to pre-eclampsia spans multiple study designs, geographic populations, and clinical timepoints. A defining characteristic of this emerging evidence base is the pronounced heterogeneity observed not only in the magnitude of reported diagnostic performance but also in the fundamental directionality of the SII–pre-eclampsia association. The principal study characteristics and findings are summarized in Table 1.

### 4.1. First-Trimester Predictive Studies

The investigation of SII for first-trimester pre-eclampsia prediction represents a particularly attractive research direction, as early risk stratification could enable targeted surveillance protocols, and the initiation of prophylactic interventions including low-dose aspirin, lifestyle modifications, and intensified antenatal monitoring for high-risk women [30,31,32,86].

Li and colleagues conducted a large multicenter retrospective cohort study representing the most comprehensive investigation of SII and pre-eclampsia to date [59]. This study enrolled 47,480 pregnant women across multiple centers in China, collecting first-trimester complete blood count data (8–14 weeks’ gestation) and following participants for pre-eclampsia development. Among the cohort, 2489 women (approximately 5.2%) developed pre-eclampsia. The first-trimester SII values demonstrated significant elevation in women who subsequently developed pre-eclampsia compared to those who remained normotensive (median SII 614.3 × 10^9^/L versus 537.8 × 10^9^/L, *p* < 0.001). A multivariate logistic regression analysis adjusting for maternal age, body mass index, nulliparity, chronic hypertension, and diabetes mellitus revealed that women in the highest SII quartile (>682.5 × 10^9^/L) exhibited 1.21-fold increased odds of pre-eclampsia development (OR 1.21, 95% CI 1.05–1.39, *p* = 0.0078). A receiver operating characteristic (ROC) curve analysis yielded an area under the curve (AUC) of 0.587 (95% CI 0.572–0.602) for pre-eclampsia prediction using first-trimester SII alone [59]. Although the large sample size confers statistical precision, the retrospective single-ethnicity design and poor AUC limit the translational significance of this finding (GRADE certainty: Low).

Özkan and colleagues conducted a retrospective cohort study in a Turkish population comparing the first-trimester SII, systemic inflammation response index (SIRI), and pan-immune inflammation value (PIV) for pre-eclampsia prediction [80]. This study enrolled 126 women who developed pre-eclampsia without severe features, 126 who developed pre-eclampsia with severe features, and 126 normotensive controls (*n* = 378 total). First-trimester SII values were significantly elevated in women who developed pre-eclampsia without severe features compared to controls, with the ROC analysis yielding an AUC of 0.801 for predicting pre-eclampsia without severe features and an optimal cut-off of 620.59 × 10^9^/L providing a sensitivity of 81% and specificity of 67%. Notably, however, SII demonstrated a markedly inferior performance for predicting pre-eclampsia with severe features (AUC 0.535), suggesting that SII may paradoxically lose discriminatory capacity for the more clinically consequential disease subtype. Among comparator indices, PIV demonstrated an AUC of 0.774 for pre-eclampsia prediction, while SIRI yielded an AUC of 0.609 [80].

In contrast, Cevher Akdulum and colleagues conducted a case–control study examining first-trimester SII in a Turkish population and reported a finding discordant with the expected direction of association [60]. This investigation enrolled 30 women with pre-eclampsia and 100 normotensive pregnant controls. First-trimester SII values (11–14 weeks’ gestation) were significantly lower in women who developed pre-eclampsia compared to controls (median 813.7 × 10^9^/L versus 1009.8 × 10^9^/L, *p* = 0.031). The ROC analysis yielded an AUC of 0.635 (95% CI 0.519–0.752) with an optimal cut-off of 836.83 × 10^9^/L, providing a sensitivity of 40% and a specificity of 60% [60].

Seyhanli and colleagues conducted a comprehensive first-trimester study examining multiple inflammatory indices including SII, SIRI, PIV, NLR, PLR, the monocyte-to-lymphocyte ratio (MLR), and the β-hCG-to-PAPP-A ratio in 105 women who developed pre-eclampsia and 171 gestational-age-matched normotensive controls (*n* = 276) [83]. Importantly, this study found that SII could not significantly predict pre-eclampsia development. The authors explicitly reported that NLR, PLR, MLR, SII, and the β-hCG-to-PAPP-A ratio lacked the predictive capacity for pre-eclampsia. In contrast, only SIRI and PIV emerged as significant predictors, indicating that, among the composite inflammatory indices evaluated, SII was among the poorest performers [83].

Sahin and colleagues examined first-trimester inflammatory indices including SII for predicting pre-eclampsia development in a Turkish cohort comprising 192 women who developed pre-eclampsia and 159 controls [81]. Consistent with the findings of Cevher Akdulum et al. [60], this study reported a paradoxical finding: first-trimester SII values were significantly lower in women who subsequently developed pre-eclampsia (713 ± 364 versus 895 ± 635 in controls, *p* = 0.02). The authors noted that most inflammatory markers were paradoxically higher in the control group. Despite this inverse relationship, the ROC analysis yielded an AUC of 0.634 for pre-eclampsia prediction [81].

### 4.2. Studies at Time of Diagnosis

An investigation of SII at the time of pre-eclampsia diagnosis provides insights into the relationship between SII and established disease, though findings at this timepoint do not inform early prediction.

Kapci and colleagues conducted a retrospective study in an emergency department setting, comparing SII values between 40 women presenting with pre-eclampsia and 40 hypertensive pregnant women without pre-eclampsia (*n* = 80 total) [82]. Notably, the control group comprised hypertensive pregnant women rather than normotensive controls, an important distinction affecting interpretation. Paradoxically, SII values were significantly lower in the pre-eclampsia group compared to hypertensive controls (median 944.23 × 10^9^/L versus 1600.37 × 10^9^/L, *p* = 0.018). The ROC analysis yielded an AUC of 0.705 (95% CI 0.587–0.823) for distinguishing pre-eclampsia from hypertensive pregnancy, with an optimal cut-off of 758.39 × 10^9^/L providing a sensitivity of 77.5% and a specificity of 67.5%. No severity stratification was performed given the small sample size [82].

### 4.3. SII in Related Pregnancy Complications

Several studies have examined SII in relation to pre-eclampsia-associated conditions rather than pre-eclampsia prediction per se.

Gao and colleagues examined the associations between multiple systemic inflammatory biomarkers (including NLR, MLR, PLR, SII, and SIRI) and pre-eclampsia-related acute kidney injury (PE-AKI) in a large retrospective observational study [87]. This investigation included 4071 women already diagnosed with pre-eclampsia, examining which inflammatory indices predicted subsequent AKI development. Importantly, this study did not compare SII between women with and without pre-eclampsia, but rather examined SII as a predictor of renal complications among women who already had pre-eclampsia. MLR and NLR demonstrated the strongest associations with PE-AKI development [87].

İpek and colleagues investigated whether SII and other inflammatory indices could predict HELLP syndrome development in a first-trimester study enrolling 28 women who subsequently developed HELLP and 100 controls [88]. While this study provides relevant data on SII in severe pregnancy complications, its specific focus on HELLP syndrome limits its direct applicability to pre-eclampsia prediction [88].

Han and colleagues conducted a retrospective cohort study evaluating placental growth factor (PlGF), vitamin D, and the pan-immune inflammation value (PIV) as predictive biomarkers for pre-eclampsia severity in 457 pregnant individuals assessed at 16–20 weeks’ gestation [89]. This study examined PIV rather than SII, and found that PlGF (AUC 0.774), vitamin D (AUC 0.805), and PIV (AUC 0.724) demonstrated a significant predictive capacity for pre-eclampsia severity. The combination of these biomarkers improved the discriminatory capacity compared to any single marker alone, supporting the potential role of composite inflammatory indices within multiparametric prediction models [89].

### 4.4. Meta-Analyses and Systematic Reviews

Zhang and He conducted a systematic review and meta-analysis examining SII in both gestational diabetes mellitus and pre-eclampsia [85]. The meta-analysis included nine studies overall, of which five specifically evaluated SII in pre-eclampsia and four addressed gestational diabetes mellitus. Importantly, the pre-eclampsia-specific subgroup analysis yielded non-significant results; the authors’ plain language summary stated that SII values were not notably different between women with or without pre-eclampsia. In contrast, the significant association was observed for gestational diabetes mellitus. The overall analysis (across both conditions) demonstrated substantial heterogeneity (I^2^ = 89%), with subgroup analyses suggesting that the heterogeneity was partially explained by the timing of assessment, population ethnicity, and disease type. These findings highlight that the pooled meta-analytic results across pregnancy complications should be interpreted with caution, and that condition-specific analyses may yield divergent conclusions [85]. The GRADE certainty for the pooled pre-eclampsia-specific estimate from this meta-analysis is Very Low, owing to the high between-study heterogeneity (I^2^ = 89%), the predominantly retrospective constituent studies, and the non-significant pooled result.

### 4.5. Comparative Performance Against Other Inflammatory Biomarkers

The available evidence does not support the consistent superiority of SII over other composite inflammatory indices for pre-eclampsia prediction. Direct head-to-head comparisons reveal a complex picture [80,83,89,90].

Seyhanli and colleagues found that SII was non-significant for pre-eclampsia prediction, whereas SIRI and PIV demonstrated a significant discriminatory capacity [83]. This finding directly contradicts a hypothesized advantage of three-component over two-component indices and suggests that SII may in fact be inferior to SIRI and PIV in certain populations.

Özkan and colleagues reported that first-trimester SII achieved an AUC of 0.801 for predicting pre-eclampsia without severe features, while PIV demonstrated an AUC of 0.774 and SIRI yielded an AUC of 0.609 [80]. However, for predicting pre-eclampsia with severe features—the more clinically relevant outcome—the SII performance declined dramatically to an AUC of 0.535, whereas SIRI maintained an AUC of 0.701, substantially outperforming SII for this critical subtype [80].

Meta-analyses comparing NLR and PLR for pre-eclampsia prediction provide additional context. Kang and colleagues pooled data from multiple NLR studies, reporting a summary AUC of 0.73 (95% CI 0.69–0.77) [46]. Zheng and colleagues reported similar pooled NLR diagnostic performance with an AUC of 0.74 [47]. For PLR, Ye and colleagues reported a pooled AUC of 0.68 (95% CI 0.64–0.72) [91]. Individual SII studies report AUC values ranging widely from 0.535 to 0.801 [59,60,80,81,82]; however, the only available meta-analysis found the pre-eclampsia-specific pooled SII analysis to be non-significant [85], precluding a meaningful comparison of the pooled estimates across indices.

Mészáros and colleagues specifically examined first-trimester NLR for pre-eclampsia screening, reporting a potential biochemical marker but highlighting the substantial inter-study heterogeneity [48]. More recent studies by Zhu and colleagues evaluating the pan-immune inflammation value (PIV) have demonstrated a dose-dependent pre-eclampsia prediction [90]. Han and colleagues similarly found that PIV, combined with PlGF and vitamin D, showed a promising predictive capacity for pre-eclampsia severity [89]. Taken together, the comparative evidence suggests that PIV and SIRI may perform comparably to or better than SII for pre-eclampsia prediction, particularly for severe disease subtypes.

### 4.6. SII in Different Populations

The available evidence spans multiple geographic and ethnic populations, predominantly from China, Turkey, and Georgia, with limited data from European and North American populations [59,60,80,81,82,83]. This geographic concentration introduces potential concerns regarding generalizability, as baseline hematological parameters and SII values may differ across ethnic groups [85,92]. Bai and colleagues established gestational age-specific reference intervals for SII during normal pregnancy in a Chinese population, demonstrating that SII values follow characteristic physiological patterns during pregnancy with increases during the third trimester [92]. Population-specific reference ranges and cut-off values may therefore be necessary for optimal clinical implementation [85,92].

Maziashvili and colleagues examined the influence of maternal age on inflammatory marker levels including SII in a study of 63 women with pre-eclampsia and 63 controls [84]. The authors reported first-trimester SII measurements and found that SII values differed significantly by age group, with women aged 26–35 demonstrating elevated values. However, no overall significant difference in SII between pre-eclampsia and control groups was reported, and no ROC analysis for SII predictive performance was performed [84]. Miller and colleagues examined diet quality and hypertensive disorders in an Asian and Pacific Islander cohort, noting that inflammatory markers varied by ethnic background [93]. These population-specific variations underscore the need for validation studies across diverse populations before establishing universal screening recommendations.

Table 2 synthesizes the key findings and strength of association across the principal studies evaluating SII in pre-eclampsia prediction and diagnosis.

**Table 1:** Key studies on SII and pre-eclampsia: characteristics and findings.

First Author (Year)	Country	Study Design	Sample Size	SII Assessment Timing	Diagnostic Performance	Key Findings	Confounders Controlled
Li et al. (2025) [59]	China	Retrospective cohort	47,480 pregnant women (2489 PE)	First trimester (8–14 weeks)	AUC 0.587	Highest SII quartile: OR 1.21 (95% CI 1.05–1.39) for PE	Age, BMI, nulliparity, chronic HTN, DM
Cevher Akdulum et al. (2023) [60]	Turkey	Case–control	30 PE cases, 100 controls	First trimester (11–14 weeks)	AUC 0.635 (0.519–0.752); Sens 40%; Spec 60%	SII significantly lower in PE (813.7 vs. 1009.8; *p* = 0.031); cut-off 836.83	Age, GA at delivery
Seyhanli et al. (2024) [83]	Turkey	Case–control	105 PE cases, 171 controls	First trimester (11–14 weeks)	SII non-significant for PE prediction	SII could not predict PE; only SIRI and PIV significant	Age, nulliparity, BMI, chronic conditions
Sahin et al. (2025) [81]	Turkey	Retrospective cohort	192 PE cases, 159 controls	First trimester	AUC 0.634	SII significantly lower in PE (713 ± 364 vs. 895 ± 635; *p* = 0.02)	Age, BMI, parity
Kapci et al. (2024) [82]	Turkey	Retrospective cohort	40 PE, 40 hypertensive controls	At diagnosis (emergency department)	AUC 0.705 (0.587–0.823); Sens 77.5%; Spec 67.5%	SII significantly lower in PE vs. hypertensive controls (944.23 vs. 1600.37; *p* = 0.018); cut-off 758.39	GA, maternal age
Özkan et al. (2024) [80]	Turkey	Retrospective cohort	126 PE, 126 PE-SF, 126 controls	First trimester	AUC 0.801 (PE without SF); Sens 81%; Spec 67%	SII higher in PE; cut-off 620.59; PE-SF AUC only 0.535; PIV AUC 0.774; SIRI AUC 0.609	Age, nulliparity, BMI, GA
Gao et al. (2025) [87]	China	Retrospective observational	4071 women with PE	At diagnosis	PE-AKI prediction; MLR and NLR strongest associations	Study of PE- related AKI, not PE prediction; MLR and NLR outperformed SII	Age, GA, PE severity
Han et al. (2025) [89]	China	Retrospective cohort	457 pregnant women (101 mild PE, 67 severe PE, 217 controls, 72 validation)	Second trimester (16–20 weeks)	PIV AUC 0.724; PlGF AUC 0.774; Vit D AUC 0.805	Evaluated PIV (not SII); combination of PlGF, Vit D, and PIV improved severity prediction	Age, BMI, parity, GA
İpek et al. (2023) [88]	Turkey	Retrospective cohort	28 HELLP, 100 controls	First trimester	–	Focus on HELLP prediction, not PE	Age, BMI, parity
Zhang and He (2025) [85]	Multi-country	Systematic review and meta-analysis	9 studies total (5 PE, 4 GDM)	Variable	PE subgroup analysis: SII not notably different in PE; I^2^ = 89%	5 of 9 studies addressed PE; non-significant PE-specific finding; significant for GDM only	N/A (meta-analysis)
Maziashvili et al. (2023) [84]	Georgia	Retrospective cohort	63 PE cases, 63 controls	First trimester	No ROC analysis performed for SII	SII elevated in 26–35 age group only; no overall significant PE difference	Age, BMI, parity, GA

PE, pre-eclampsia; PE-SF, pre-eclampsia with severe features; AUC, area under the curve; Sens, sensitivity; Spec, specificity; BMI, body mass index; GA, gestational age; HTN, hypertension; DM, diabetes mellitus; AKI, acute kidney injury; NLR, neutrophil-to-lymphocyte ratio; PLR, platelet-to-lymphocyte ratio; MLR, monocyte-to-lymphocyte ratio; SIRI, systemic inflammation response index; PIV, pan-immune inflammation value; PE-AKI, pre-eclampsia-related acute kidney injury.

**Table 2:** Summary of SII association with pre-eclampsia across principal studies.

Study (Year)	Study Design	Sample Size	Key Findings	Direction and Strength of Association
Li et al. (2025) [59]	Retrospective cohort	47,480 pregnancies	Highest SII quartile: OR 1.21 (95% CI 1.05–1.39) for PE; AUC 0.587	SII elevated in PE; significant but modest
Cevher Akdulum et al. (2023) [60]	Case–control	130 women	SII lower in PE (813.7 vs. 1009.8; *p* = 0.031); AUC 0.635	SII decreased in PE; inverse association
Seyhanli et al. (2024) [83]	Case–control	276 women	SII non-significant for PE prediction; only SIRI and PIV significant	No significant association; SII could not predict PE
Sahin et al. (2025) [81]	Retrospective cohort	351 women	SII lower in PE (713 ± 364 vs. 895 ± 635; *p* = 0.02); AUC 0.634	SII decreased *n* PE; inverse association
Kapci et al. (2024) [82]	Retrospective cohort	80 women (PE vs. hypertensive controls)	SII lower in PE vs. hypertensive controls (944.23 vs. 1600.37; *p* = 0.018); AUC 0.705	SII decreased in PE; inverse association
Özkan et al. (2024) [80]	Retrospective cohort	378 women	SII elevated in PE without SF (AUC 0.801); AUC only 0.535 for PE with SF	SII elevated in PE without SF; non- discriminatory for severe PE
Zhang and He (2025) [85]	Meta-analysis	9 studies (5 PE, 4 GDM)	PE subgroup analysis non-significant; I^2^ = 89%	Non-significant pooled PE association

## 5. Discussion

The findings from this literature review reveal that the relationship between SII and pre-eclampsia is substantially more complex and heterogeneous than initially hypothesized. Rather than demonstrating a consistent pattern of SII elevation in pre-eclampsia, the available evidence presents conflicting directionality, variable diagnostic performance, and non-significant pooled meta-analytic results, all of which necessitate careful interpretation.

### 5.1. Interpretation of Key Findings

The most striking finding of this review is the heterogeneity in the direction of the SII–pre-eclampsia association across studies. Of the six studies directly comparing SII values between pre-eclampsia and control groups, only two (Li et al. [59] and Özkan et al. [80]) reported the expected pattern of SII elevation in women with pre-eclampsia. Three studies (Cevher Akdulum et al. [60], Sahin et al. [81], and Kapci et al. [82]) reported significantly lower SII values in the pre-eclampsia group, while one study (Seyhanli et al. [83]) found no significant difference. The meta-analysis by Zhang and He [85], which pooled five pre-eclampsia-specific studies, reported non-significant results, consistent with this pattern of conflicting individual findings.

This directional heterogeneity has important implications for the theoretical framework underlying SII application in pre-eclampsia. While the biological rationale predicts SII elevation due to neutrophil activation, platelet changes, and lymphocytopenia, the paradoxical decrease observed in some studies may reflect the complex and variable nature of hematological changes in pre-eclampsia. Platelet consumption (thrombocytopenia) in advanced disease may reduce the SII numerator, while compensatory bone marrow responses may increase lymphocyte production, both of which would lower SII values. The timing of the assessment, disease phenotype (early-onset versus late-onset), severity classification, and population-specific hematological baselines may all contribute to the observed heterogeneity. A structured examination of potential heterogeneity sources reveals several important patterns. With respect to the clinical context, the distinction between predictive studies (first-trimester assessment in prospectively followed cohorts) and diagnostic studies (SII measured at the time of established pre-eclampsia) is critical yet rarely accounted for in the existing evidence base. First-trimester predictive studies span a wide performance range (AUC 0.535–0.801) and include the two studies reporting paradoxically lower SII values [60,81], while the single diagnostic study (Kapci et al. [82]) similarly identified a lower SII in pre-eclampsia relative to hypertensive controls—a finding that may reflect the consumptive thrombocytopenia intrinsic to established disease rather than any predictive deficit. These two clinical contexts are not directly comparable and should not be conflated in evidence synthesis (GRADE certainty: Very Low). With respect to the disease severity, the available data are particularly instructive: Özkan and colleagues [80] demonstrated that SII discriminated between pre-eclampsia without severe features with an AUC of 0.801, yet performed no better than the chance for pre-eclampsia with severe features (AUC of 0.535). This severity-dependent performance inversion is plausibly explained by the consumptive thrombocytopenia characteristic of severe disease, which reduces the SII numerator and offsets any neutrophilia-driven elevation. With respect to the gestational timing, all primary studies assessed SII in the first trimester (8–14 weeks), precluding conclusions regarding the second- or third-trimester predictive performance. The meta-analysis by Zhang and He [85] noted that the timing of the assessment contributed substantially to the between-study heterogeneity (I^2^ = 89%), reinforcing the view that pooled estimates across heterogeneous timepoints are unreliable (GRADE certainty: Very Low).

Regarding the diagnostic performance, individual study AUC values range from 0.535 to 0.801, spanning from non-discriminatory to moderate performance [59,60,80,81,82]. The largest study (Li et al., *n* = 47,480) reported an AUC of only 0.587, indicating a poor discriminatory capacity [59]. Özkan and colleagues reported the highest AUC of 0.801, but this applied only to pre-eclampsia without severe features, with performance dropping dramatically to an AUC of 0.535 for pre-eclampsia with severe features [80]. This differential performance is clinically concerning, as it suggests SII may be least useful precisely where prediction would be most valuable—for severe disease requiring urgent intervention.

The sensitivity and specificity values reported across studies (a sensitivity of 40–81%, and a specificity of 60–67.5%) indicate substantial false-positive and false-negative rates that would severely limit standalone clinical utility [60,80,82].

### 5.2. Biological and Pathophysiological Mechanisms

Multiple physiological pathways potentially mediate the association between SII and pre-eclampsia, though the inconsistent directionality of this association complicates mechanistic interpretation. The neutrophil component reflects the excessive inflammatory activation and oxidative stress generation characteristic of pre-eclampsia pathophysiology [61,62,63,64,65]. Neutrophil-derived NETs contribute to endothelial damage, platelet activation, and thrombotic microangiopathy, processes central to pre-eclampsia end-organ damage [62,68,69]. The lymphocyte component captures the immune regulatory deficits fundamental to disease pathogenesis, including Treg depletion and Th1/Th17 expansion [36,37,38,39,68,69]. The platelet component reflects the prothrombotic state and enhanced platelet–leukocyte interactions that amplify inflammatory cascades [56,57,75,76,77,78].

However, the paradoxical decrease in SII observed in three studies [60,81,82] suggests that the interplay between SII components may be more complex than the theoretical framework implies. In pre-eclampsia, consumptive thrombocytopenia—a hallmark of severe disease—could reduce the platelet component of the SII numerator, potentially offsetting neutrophil elevation. Similarly, the relative contributions of neutrophilia versus lymphocytopenia may vary with the disease phenotype, gestational age, and timing of assessment. Kapci et al.’s finding that SII was lower in pre-eclampsia compared to hypertensive controls [82] further suggests that hypertension-associated inflammatory changes may independently elevate SII, complicating interpretation when disease-specific patterns are sought. Studies examining individual SII components alongside the composite index across different disease stages and phenotypes are urgently needed in order to address these mechanistic questions.

### 5.3. Comparison with Established Prediction Methods

When compared to established pre-eclampsia prediction methods, SII demonstrates substantially inferior and inconsistent performance. The FMF first-trimester combined screening algorithm, integrating maternal factors, mean arterial pressure, uterine artery Doppler, and PlGF, achieves detection rates of approximately 90% for early-onset pre-eclampsia and 75% for preterm pre-eclampsia at a 10% false-positive rate [24,27,28]. The sFlt-1/PlGF ratio demonstrates AUC values exceeding 0.90 for short-term pre-eclampsia prediction in symptomatic women [16,25,26]. In comparison, SII AUC values of 0.535–0.801 across individual studies [59,60,80,81,82], with non-significant pooled meta-analytic results [85], clearly preclude SII as a replacement for these established approaches. The clinical significance of these AUC values warrants explicit contextualization. In obstetric screening, a clinically useful first-trimester biomarker is generally expected to achieve an AUC ≥ 0.75–0.80, with a sensitivity ≥ 75% at a fixed 10% false-positive rate—thresholds derived from the FMF competing-risks model performance benchmarks [27,28]. By this standard, only the AUC of 0.801 reported by Özkan et al. [80] approaches clinical utility, and this applied exclusively to pre-eclampsia without severe features. The AUC of 0.587 from the largest study (Li et al. [59], *n*  =  47,480) corresponds to a positive likelihood ratio of approximately 1.2, indicating essentially no clinically meaningful risk stratification. For a screening test intended to guide aspirin prophylaxis initiation—where correct identification before 16 weeks is the critical therapeutic window [30,31]—such discriminatory capacity is inadequate. These considerations substantially reinforce the conclusion that SII, in its current evidence status, has no established role in clinical pre-eclampsia screening (GRADE certainty: Very Low).

A comparison with other composite inflammatory indices yields a similarly nuanced picture. While NLR meta-analyses report pooled AUC values of 0.73–0.74 [46,47] and PLR of approximately 0.68 [91], SII has not achieved comparable pooled estimates, as the only available meta-analysis found non-significant pre-eclampsia-specific results [85]. In direct head-to-head comparisons, SII’s performance relative to other novel composite indices is inconsistent: Seyhanli et al. found SII to be non-significant while SIRI and PIV were significant predictors [83], and Özkan et al. showed that SIRI substantially outperformed SII for predicting pre-eclampsia with severe features (AUC 0.701 versus 0.535) [80]. PIV has emerged as a potentially superior alternative, with Zhu and colleagues demonstrating a dose-dependent pre-eclampsia prediction [90] and Han and colleagues reporting a PIV AUC of 0.724 within a multiparametric model [89].

### 5.4. Population-Specific Considerations

The effectiveness of SII for pre-eclampsia prediction may vary across different population groups, influenced by factors such as ethnicity, baseline hematological parameters, dietary patterns, and prevalent comorbidities [84,85,92,93]. The current evidence base is geographically concentrated in East Asian, Middle Eastern, and Caucasian populations, with limited data from African, South American, and North American cohorts [59,60,80,81,82,83,84]. This geographic concentration raises concerns about generalizability, as baseline SII values and physiological ranges during pregnancy may differ across ethnic groups [92]. Beyond inter-ethnic biological variability, pre-analytical and analytical sources of variation represent a largely overlooked yet potentially important contributor to between-study heterogeneity. SII is derived from three complete blood count parameters—neutrophil, platelet, and lymphocyte counts—each susceptible to multiple pre-analytical influences. The fasting status at the time of sampling may affect neutrophil and lymphocyte counts through postprandial immune activation; concurrent subclinical or overt infections predictably elevate neutrophil counts and thereby inflate SII independent of any pre-eclamptic process. Corticosteroid administration, which profoundly alters leukocyte trafficking patterns, and anticoagulant therapy, which may affect platelet measurement, represent additional confounders inconsistently reported or controlled across included studies. At the analytical level, the hematology analyzer type, calibration standards, and the specific EDTA concentration of the collection tube can introduce systematic inter-laboratory variation in platelet and differential leukocyte counts. None of the primary studies reported standardized pre-analytical protocols, and this absence of methodological harmonization constitutes a fundamental barrier to cross-study comparability that prospective SII validation studies must explicitly address [85,94].

Bai and colleagues established gestational age-specific reference intervals for SII during normal pregnancy in a Chinese population, demonstrating characteristic physiological patterns, with SII values increasing during the third trimester [92]. Population-specific reference ranges and optimal cut-off values may therefore be necessary, analogous to the ethnic-specific adjustment factors employed in established screening algorithms [24,27,85]. The substantial variability in the reported optimal cut-off values across existing studies (ranging from 620.59 to 836.83 × 10^9^/L for the first-trimester assessment) [60,80] likely reflects both population differences and methodological variability in threshold determination. Notably, the inconsistent direction of association across studies further undermines the feasibility of establishing universal cut-off thresholds.

### 5.5. Integration with Other Predictive Strategies

Given pre-eclampsia’s multifactorial nature and SII’s inconsistent performance as a standalone biomarker, integration within comprehensive multiparametric prediction models represents the most promising potential clinical application [24,27,28,89,95]. Contemporary best-practice pre-eclampsia prediction employs combined algorithms incorporating maternal demographic and clinical characteristics, biophysical measurements, and biochemical markers [24,27,28]. Whether an SII addition to such comprehensive models provides an incremental predictive value beyond the information already captured by these established parameters remains unexplored [89,95].

Han and colleagues’ finding that combining PIV with PlGF and vitamin D improved the discriminatory capacity for severe pre-eclampsia provides preliminary evidence supporting the multiparametric integration of composite inflammatory indices, though this study evaluated PIV rather than SII specifically [89]. A formal evaluation through nested model comparison, net reclassification improvement, and integrated discrimination improvement analyses is required in order to determine whether SII provides true incremental value [95,96]. In the context of obstetric screening, clinically meaningful incremental value from the addition of a new biomarker to an established model is typically defined as a net reclassification improvement (NRI) exceeding 5–10% or an integrated discrimination improvement (IDI) that translates to a measurable change in the clinical action rate—such as a change in the proportion of women correctly identified for aspirin prophylaxis initiation [95,96]. These metrics are substantially more informative than simple AUC comparisons, which are notoriously insensitive to incremental discrimination when a baseline model already performs adequately [96]. Importantly, since NLR already captures neutrophil-to-lymphocyte dynamics and is derivable from the same complete blood count as SII, the marginal information contributed by additionally incorporating the platelet count into the SII formula must be explicitly quantified rather than assumed. No existing study has performed such a formal incremental analysis for SII within established pre-eclampsia screening algorithms. Until such evidence is generated, the theoretical complementarity of SII with placental biomarkers remains speculative and cannot justify its inclusion in clinical prediction pathways. The potential complementarity of composite inflammatory indices—reflecting the systemic inflammatory status—with placental biomarkers—reflecting placental dysfunction—is biologically plausible and warrants rigorous investigation, though the current evidence for SII specifically is insufficient to support its inclusion in prediction models [16,49,89].

### 5.6. Clinical Implications and Practice Recommendations

Based on the current evidence, SII cannot be recommended for clinical use in pre-eclampsia prediction or diagnosis. The conflicting directionality of the SII–pre-eclampsia association, non-significant meta-analytic results, and inconsistent diagnostic performance across studies preclude evidence-based clinical implementation. While SII’s derivation from routine complete blood count testing presents the theoretical advantages of cost-effectiveness and universal accessibility, these practical benefits are meaningless without a reliable and reproducible predictive performance [59,85].

In the event that future prospective studies demonstrate consistent associations, standardized SII calculation protocols, population-specific or gestational-age-specific reference ranges, and validated cut-off thresholds would be prerequisite for any clinical application [85,92]. The integration of SII calculation into automated laboratory information systems could facilitate seamless implementation without additional clinical workflow burden. Clinicians should be aware that SII is influenced by numerous conditions beyond pre-eclampsia, including infections, hematological disorders, and other inflammatory states, necessitating clinical correlation [49,50,94].

### 5.7. Limitations of Current Evidence

The evidence base for SII in pre-eclampsia prediction has significant limitations that must be acknowledged. The predominance of retrospective study designs (all primary studies are retrospective) introduces potential selection, information, and temporal biases that prospective studies would mitigate [59,60,81,82,83,87]. The small sample sizes in most studies (with the notable exception of the Li et al. multicenter cohort), particularly in the studies reporting inverse associations (Cevher Akdulum et al., *n* = 130; Kapci et al., *n* = 80), limit the statistical power and precision of diagnostic performance estimates [60,82].

Study heterogeneity—including diverse populations, varying pre-eclampsia diagnostic criteria, inconsistent SII assessment timing, different control group definitions (normotensive versus hypertensive), and different statistical approaches—complicates direct comparison and evidence synthesis [85]. The absence of standardized, externally validated cut-off values is a critical limitation; optimal thresholds vary substantially across studies, and most were derived from the same cohorts used for evaluation, introducing optimistic performance estimates [60,80]. Furthermore, most studies lack a formal assessment of SII’s incremental value when added to established prediction models, leaving uncertain whether SII provides information beyond what clinical risk factors and conventional biomarkers already capture [89,95].

The limited information on potential confounders including maternal infections, autoimmune conditions, hematological disorders, and medication use that may influence SII values represents another important limitation [85,94]. Finally, no intervention trials have evaluated whether SII-guided clinical management improves maternal or perinatal outcomes compared to standard care, the ultimate criterion for biomarker clinical utility [96].

### 5.8. Future Research Directions

Several critical research priorities emerge from this review. Large-scale, multicenter prospective cohort studies with a standardized methodology across diverse populations are urgently needed in order to resolve the fundamental question of SII directionality in pre-eclampsia and to validate or refute its predictive performance [85,92,95]. Studies should explicitly examine whether the direction of SII association differs by pre-eclampsia subtype (early-onset versus late-onset, with versus without severe features), gestational timing of assessment, and population characteristics. Integration studies formally evaluating SII’s incremental value within established multiparametric prediction models (e.g., the FMF algorithm) would determine whether SII adds clinically meaningful information [24,27,89,95]. Longitudinal trajectory analyses examining serial SII measurements throughout pregnancy may reveal superior predictive patterns compared to a single-timepoint assessment [92,97]. Mechanistic investigations examining the correlations between SII and circulating NET biomarkers, regulatory T-cell frequencies, angiogenic factor ratios, and specific cytokine profiles would strengthen our biological understanding and may explain the observed directional heterogeneity [62,68,69,70,71]. Ultimately, randomized controlled trials evaluating whether SII-guided clinical management improves outcomes are prerequisite for evidence-based clinical implementation [96].

## 6. Conclusions

This narrative review provides a critical appraisal of the Systemic Immune-Inflammation Index as a candidate biomarker for pre-eclampsia prediction and diagnosis. Although SII’s simultaneous capture of neutrophil activation, lymphocyte dysfunction, and platelet alterations offers a theoretically compelling composite measure of the systemic inflammatory and immune dysregulation central to pre-eclampsia pathogenesis, the available empirical evidence does not consistently substantiate this premise. The current evidence base is characterized by substantial heterogeneity, both in the directionality of the SII–pre-eclampsia association and in the reported diagnostic performance (AUC pf 0.535–0.801). The only available meta-analysis yielded non-significant pooled results for the pre-eclampsia-specific subgroup, and the largest individual study demonstrated a poor discriminatory capacity. While SII’s derivation from universally obtained, zero-incremental-cost complete blood count parameters represents a meaningful practical advantage, this alone is insufficient to justify clinical implementation in the context of inconsistent directionality, the absence of standardized thresholds, and the predominance of small retrospective studies. At present, SII must be regarded as a strictly exploratory biomarker whose potential future clinical role, if any, is confined to externally validated multiparametric prediction models—not as a standalone screening tool. Future research should prioritize large-scale prospective validation studies across diverse populations and pre-eclampsia subtypes, mechanistic investigations, and formal integration analyses within established multiparametric prediction frameworks, applying rigorous incremental value metrics (NRI, and IDI). The parallel evaluation of alternative composite inflammatory indices, particularly PIV and SIRI, which demonstrated a superior performance in available head-to-head comparisons, is similarly warranted.

## Data Availability

No new data were created or analyzed in this study. Data sharing is not applicable to this article.
